# A TALE OF TWO CASE STUDIES: ACCELERATED RESOLUTION THERAPY FOR COMPLICATED GRIEF IN OLDER ADULTS

**DOI:** 10.1093/geroni/igz038.1010

**Published:** 2019-11-08

**Authors:** Harleah G Buck, Diego F Hernandez, Tina Mason, Cindy Tofthagen, Kevin E Kip

**Affiliations:** 1 University of South Florida, Tampa, Florida, United States; 2 University of South Florida, Tampa, United States; 3 H.L. Moffitt Cancer Center, Tampa, Florida, United States; 4 Mayo Clinic, Jacksonville, Florida, United States

## Abstract

Complicated grief (CG) is characterized by lengthy, intense, and functionally impairing grief which disproportionately affects older adults. Accelerated Resolution Therapy (ART) is a brief, protocol driven, exposure/imagery rescripting therapy which uses lateral left-right eye movements. ART, unlike traditional psychotherapy, directs the person to perform two tasks simultaneously (e.g. re-experiencing the grief experience and performing eye movements), taxing limited working memory capacity. Importantly, this may force memory traces representing events, emotions, and sensations to compete for permanence, as well as reduce the vividness and emotional intensity of the original grief. Two CG case studies are presented (expected; unexpected death) with their response to ART. Stake’s instrumental case study methodology was used to identify and study cases which reflect a range of CG. Additionally, CG was measured by the Inventory of Complicated Grief (ICF). ICF’s range is 0-76 with scores > 24 indicating CG. Case 1 was a spousal caregiver with a single, expected death where helplessness, guilt, shame, and a life alone had resulted in CG (baseline ICF 33). Her ICF at 8 weeks post-ART was 10. Case 2 was an adult child caregiver with multiple (parent, sibling), unexpected deaths in quick succession where loss, guilt, anger, and helplessness had resulted in CG (baseline ICF 25). Her ICF at 8 weeks post-ART was 9. Both participants were able to process the distressing sensations that emerged during the imaginal exposure component facilitated with the use of eye movements. This suggests that ART may be a powerful new mind-body treatment for CG.

